# m^6^A methylation controls pluripotency of porcine induced pluripotent stem cells by targeting SOCS3/JAK2/STAT3 pathway in a YTHDF1/YTHDF2-orchestrated manner

**DOI:** 10.1038/s41419-019-1417-4

**Published:** 2019-02-20

**Authors:** Ruifan Wu, Youhua Liu, Yuanling Zhao, Zhen Bi, Yongxi Yao, Qing Liu, Fengqin Wang, Yizhen Wang, Xinxia Wang

**Affiliations:** College of Animal Sciences, Zhejiang University, Key Laboratory of Animal Nutrition & Feed Sciences, Ministry of Agriculture, Zhejiang Provincial Laboratory of Feed and Animal Nutrition, No. 866 Yuhangtang Road, Hangzhou, Zhejiang 310058 China

## Abstract

Embryonic stem cells (ESCs) and induced pluripotent stem cells (iPSCs) hold great promise for regenerative medicine, disease treatment, and organ transplantation. As the ethical issue of human ESCs and similarity of pig in human genome and physiological characteristics, the porcine iPSCs (piPSCs) have become an ideal alternative study model. *N*^6^-methyladenosine (m^6^A) methylation is the most prevalent modification in eukaryotic mRNAs, regulating the self-renewal and differentiation of pluripotency stem cells. However, the explicit m^6^A-regulating machinery remains controversial. Here, we demonstrate that m^6^A modification and its modulators play a crucial role in mediating piPSCs pluripotency. In brief, loss of METTL3 significantly impairs self-renewal and triggers differentiation of piPSCs by interfering JAK2 and SOCS3 expression, further inactivating JAK2–STAT3 pathway, which then blocks the transcription of KLF4 and SOX2. We identify that both of JAK2 and SOSC3 have m^6^A modification at 3′UTR by m^6^A-seq analysis. Dual-luciferase assay shows that METTL3 regulates JAK2 and SOCS3 expression in an m^6^A-dependent way. RIP-qPCR validates JAK2 and SOCS3 are the targets of YTHDF1 and YTHDF2, respectively. SiMETTL3 induced lower m^6^A levels of JAK2 and SOCS3 lead to the inhibition of YTHDF1-mediated JAK2 translation and the block of YTHDF2-dependent SOCS3 mRNA decay. Subsequently, the altered protein expressions of JAK2 and SOCS3 inhibit JAK2–STAT3 pathway and then the pluripotency of piPSCs. Collectively, our work uncovers the critical role of m^6^A modification and its modulators in regulating piPSCs pluripotency and provides insight into an orchestrated network linking the m^6^A methylation and SOCS3/JAK2/STAT3 pathway in pluripotency regulation.

## Introduction

Embryonic stem cells (ESCs) offer great hope for regenerative medicine, organ transplantation, and drug development. These cells also provide a powerful model system for studies of cellular identity and early mammalian development^1^. However, there are ethical issues regarding destroying human embryos and fetuses for cells isolation. The pig is an excellent model for human disease and clinical medicine applications, because of similarity in human genome and physiological characteristics^2,3^. Nevertheless, no authentic porcine embryonic stem cells (pESCs) have been isolated successfully. Induced pluripotent stem cell (iPSCs) are a type of embryonic stem cell-like pluripotent stem cell that has indefinite self-renewal and could differentiate into all types of cells^4^. Moreover, iPSCs and ESCs and are extremely similar in morphology, gene and protein expression, differentiation ability, and epigenetic modification status. Therefore, porcine induced pluripotent stem cells (piPSCs) now become an ideal alternative resource, which holds unprecedented promise for human regenerative medicine, disease treatment, and organ transplantation. However, the mechanisms of porcine embryonic development and the pluripotent regulation network remain largely unknown.

Epigenetic regulation has been elucidated to play an important role in manipulating stem cell fate^5,6^. *N*^6^-methyladenosine (m^6^A) methylation, the most prevalent internal modification in mammalian mRNAs, is widely conserved in eukaryotic species that range from yeast to humans^7–10^. The m^6^A modification is post-transcriptionally installed by the methyltransferase complex (METTL3, METTL14, and WTAP), reversed by demethylases (FTO, ALKBH5) and recognized by m^6^A-binding proteins (YTHDF1-3, YTHDC1, 2). At the molecular level, this dynamic epigenetic modification has been demonstrated to regulate RNA stability, translation, alternative splicing, and nuclear export^11–14^.

Recent studies have revealed a crucial role for m^6^A methylation and METTL3 in regulating the pluripotency and differentiation of stem cells^15–18^. Nevertheless, the function of m^6^A modification in ESCs has been investigated with discrepant results among different studies. One model reported that m^6^A modification destabilizes developmental regulators and maintains pluripotency^15^. Other studies proposed that m^6^A is not required for ESC maintenance but for cell fate transition of ESCs to differentiated lineages^16,17^. Thus, the explicit biological role of m^6^A modification in self-renewal and differentiation of pluripotency stem cell remains to be elucidated.

In the present study, we provide strong evidence for the vital role of m^6^A and its modulators in maintaining self-renewal and pluripotency of piPSCs. We demonstrate that METTL3 depletion significantly impairs self-renewal and triggers differentiation of piPSCs by inactivating JAK2–STAT3 pathway. Further study shows that METTL3 regulates JAK2–STAT3 pathway by mediating the expression SOCS3 (a negative regulator of JAK2–STAT3) and JAK2 in an m^6^A-YTHDF1/YTHDF2-dependent manner. For the first time, our findings illustrate an orchestrated network linking the m^6^A methylation and SOCS3/JAK2/STAT3 pathway in pluripotency regulation.

## Results

### METTL3 is required for piPSCs self-renewal and pluripotency

**Fig. 1:**
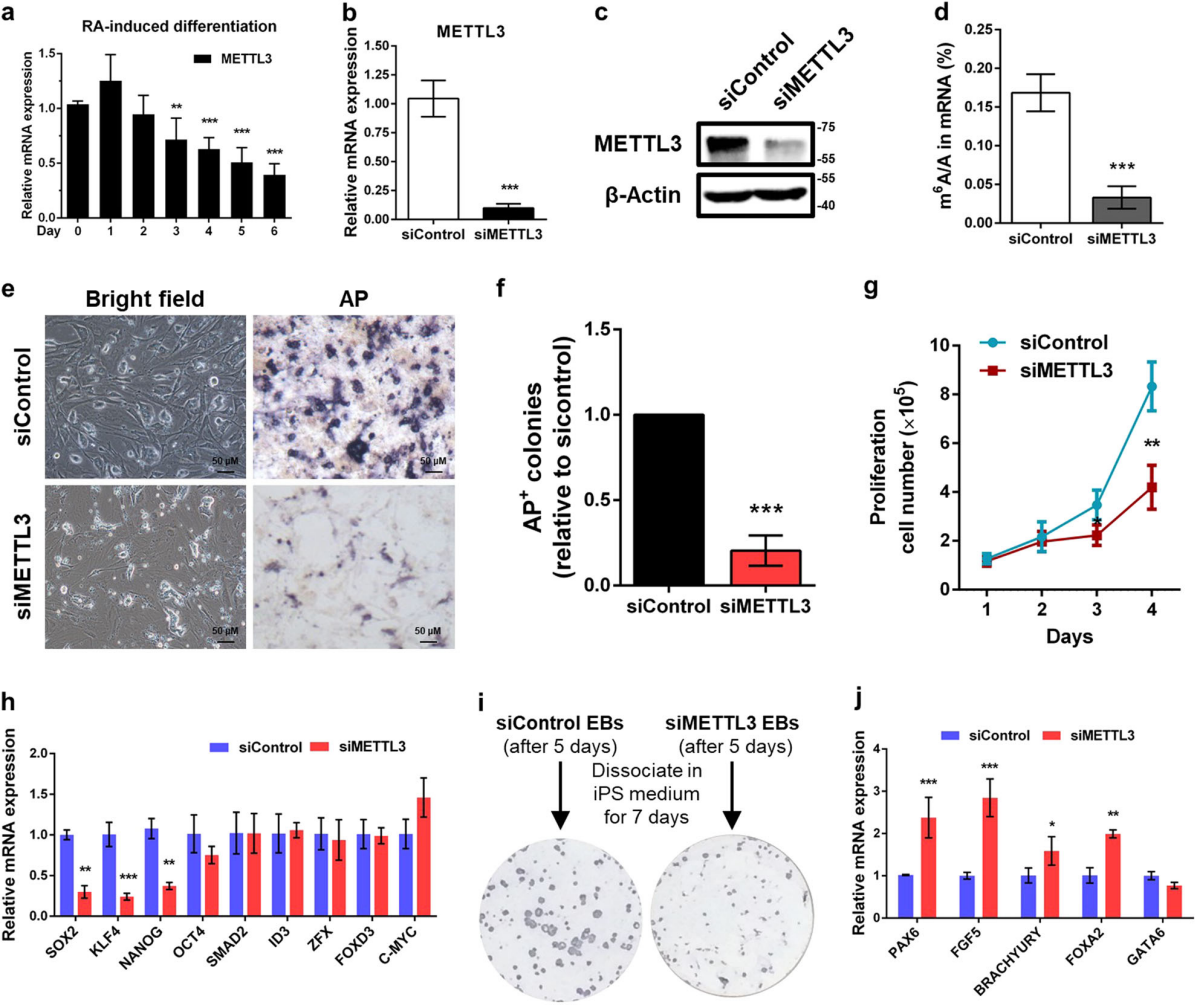
METTL3 is required for piPSCs self-renewal and pluripotency. **a** Real-time quantitative PCR (qPCR) analysis of METTL3 expression in piPSCs during RA-induced differentiation. GAPDH was used as an internal control. **b**, **c** METTL3 knockdown efficiency was measured by qPCR **b** and western blot **c**. For the immunoblot, β-actin was used as loading control. **d** Liquid chromatography-tandem mass spectrometry (LC-MS/MS) quantification of the m^6^A/A ratio in mRNA from piPSCs with or without METTL3 knockdown. **e** Morphology and Alkaline phosphatase (AP) staining of piPSCs with or without METTL3 knockdown. **f** Quantification of AP-positive colonies of piPSCs with or without METTL3 knockdown. **g** Cell proliferation assay of piPSCs with or without METTL3 knockdown. **h** qPCR analysis of SOX2, KLF4, NANOG, OCT4, SMAD2, ID3, ZFX, FOXD3, and C-MYC expression in piPSCs with or without METTL3 knockdown. **i** AP staining of EBs differentiated from piPSCs with or without METTL3 knockdown. PiPSCs with or without METTL3 knockdown were transferred to serum-based media without 2i/LIF for 5 days, to promote cell differentiation. The cells were then disaggregated, re-plated on feeder cells and grown in piPSCs conditions for 7 days. **j** qPCR analysis of PAX6, FGF5, BRACHYURY, FOXA2, and GATA6 expression in piPSCs with or without METTL3 knockdown. Data were presented as mean ± SD of three independent experiments. ^*^P < 0.05, ^**^P < 0.01, ^***^P < 0.001 compared with the control group

We first examined the m^6^A methyltransferase METTL3 expression of piPSCs in retinoic acid (RA)-induced differentiation and revealed a gradual decrease in METTL3 levels (Fig. 1a). To explore the regulatory role of METTL3 in piPSCs self-renewal and pluripotency, we next conducted loss-of-function assays by using small-interfering RNA (siRNA) that exhibited at least 90% endogenous METTL3 RNA and protein expression were inhibited in piPSCs (Fig. 1b, c). Liquid chromatography-tandem mass spectrometry (LC-MS/MS) analysis of global m^6^A level in purified mRNA from cells with or without METTL3 knockdown showed that METTL3 ablation leads to a significant reduction (~ 80%) of m^6^A on mRNA (Fig. 1d), confirming the methylation activity of METTL3 in piPSCs.

Morphologically, METTL3-depleted piPSCs colonies were flatter and less compact with significantly decreased levels of alkaline phosphatase (AP) staining relative to control colonies (Fig. 1e, f). Moreover, we found that METTL3 depletion markedly decreased the proliferation rate of piPSCs (Fig. 1g). Importantly, real-time quantitative PCR (qPCR) analysis indicated that knockdown of METTL3 decreased the gene expression of core pluripotency genes that endow stem cells with self-renewal ability, such as SRY-box 2 (SOX2), Kruppel-like factor 4 (KLF4), and Nanog homeobox (NANOG), whereas Octamer-binding transcription factor 4 (OCT4), SMAD2, ID3, ZFX, FOXD3, and C-MYC expression was unchanged (Fig. 1h), suggesting that loss of METTL3 impairs self-renewal and pluripotency in piPSCs.

To test their differentiation ability, control and METTL3-depleted piPSCs were transferred to differentiation media without 2i/LIF for embryoid bodies (EBs) for 5 days. Next, EBs were disaggregated and re-plated in piPSCs growth conditions for 7 days. AP staining revealed that only control EBs efficiently regenerated stable piPSCs (Fig. 1i). Consistently, the mRNA levels of most developmental regulators were also significantly upregulated in METTL3-deficient cells relative to control cells (Fig. 1j). Taken together, these results illuminate that METTL3 is essential to maintain the pluripotency state of piPSCs.

### METTL3 regulates piPSCs pluripotency via STAT3-KLF4-SOX2 signal axis

**Fig. 2:**
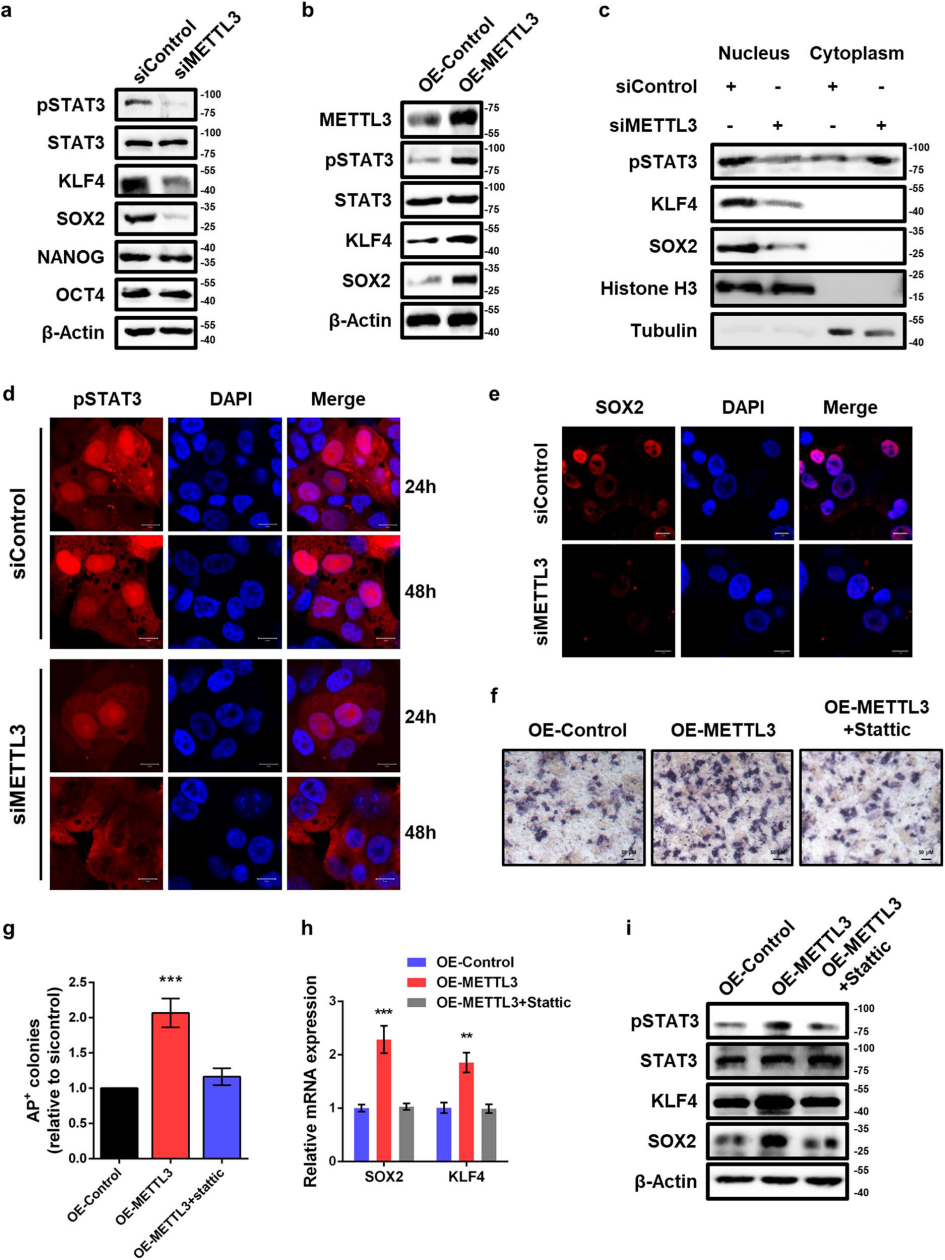
Inhibition of METTL3 impairs piPSCs pluripotency by suppressing STAT3/KLF4/SOX2 signaling. **a** Western blot analysis of pSTAT3, STAT3, KLF4, SOX2, NANOG, and OCT4 in piPSCs with or without METTL3 knockdown. β-Actin was used as loading control. **b** Western blot analysis of METTL3, pSTAT3, STAT3, KLF4, and SOX2 in piPSCs transfected with control or METTL3 plasmid. **c** Western blot of nuclear and cytoplasmic distribution of pSTAT3, KLF4, and SOX2 in piPSCs with or without METTL3 knockdown. Histone H3 and Tubulin serve as nuclear and cytoplasmic markers, respectively. **d** Immunofluorescence analysis of pSTAT3 in piPSCs transfected with siControl or siMETTL3 after 24h and 48h. Scale bar, 10 μm. **e** Immunofluorescence analysis of SOX2 in piPSCs with or without METTL3 knockdown. Scale bar, 10 μm. **f** AP staining of piPSCs transfected with control or METTL3 plasmid and treated with DMSO or 1 μm Stattic. **g** Quantification of AP-positive colonies of piPSCs transfected with control or METTL3 plasmid and treated with DMSO or 1 μm Stattic. **h** qPCR analysis of piPSCs transfected with control or METTL3 plasmid and treated with DMSO or 1 μm Stattic. GAPDH was used as an internal control. **i** Western blot analysis of pSTAT3, STAT3, KLF4, and SOX2 of piPSCs transfected with control or METTL3 plasmid and treated with DMSO or 1 μm Stattic. Data were presented as mean ± SD of three independent experiments. ^**^P < 0.01, ^***^P < 0.001 compared with the control group

It is well established that signal transducer and activator of transcription 3 (STAT3), a latent transcription factor that upon phosphorylation, has a critical role in the maintenance of embryonic stem cell pluripotency^19–21^. KLF4, a direct JAK-STAT3 downstream target, is transcriptionally activated by STAT3 phosphorylation and preferentially activates SOX2^22^. Thus, we hypothesized that loss of METTL3 downregulated gene expression of SOX2 and KLF4 by inhibiting phosphorylated STAT3 (pSTAT3). Indeed, knockdown of METTL3 significantly reduced STAT3 phosphorylation levels compared with control cells (Fig. 2a). Consistent with qPCR results, the protein expression of KLF4 and SOX2 were decreased upon METTL3 knockdown (Fig. 2a). Moreover, overexpression of METTL3 enhanced STAT3 phosphorylation and increased the protein abundance of KLF4 and SOX2 (Fig. 2b), indicating a positive correlation between METTL3 and STAT3 phosphorylation.

STAT3 is phosphorylated on the residue (Tyr-705), dimerizes and then translocates from the cytoplasm to the nucleus to activate transcription of target genes in stem cells^20^. To investigate whether METTL3 affected piPSCs pluripotency through STAT3 phosphorylation, we examined nuclear-cytoplasmic shuttling of pSTAT3 following METTL3 knockdown. As expected, we observed a dramatically decreased nuclear retention and subsequently increased cytoplasmic localization of pSTAT3 in METTL3 knockdown cells (Fig. 2c). Furthermore, the nucleic expression of KLF4 and SOX2 were repressed in METTL3 knockdown piPSCs relative to control cells (Fig. 2c). In support, immunofluorescence analysis indicated that METTL3 depletion reduced the expression of pSTAT3 in nuclear speckle (Fig. 2d). Consistently, the decreased nuclear accumulation of SOX2 was also observed (Fig. 2e).

To further confirm the role of STAT3 phosphorylation in METTL3-mediated pluripotency of piPSCs, we treated control and METTL3-overexpressed piPSCs with or without Stattic, a selective inhibitor of STAT3 phosphorylation^23^. AP staining analysis showed that forced expression of METTL3 enhanced piPSCs pluripotency, which could be effectively reversed by Stattic treatment (Fig. 2f, g). Consistently, Stattic also reversed the increased mRNA and protein levels of SOX2 and KLF4 caused by METTL3 overexpression (Fig. 2h, i). Together, our findings indicate that METTL3 maintains piPSCs pluripotency by activating STAT3-KLF4-SOX2 signaling.

### METTL3 controls the STAT3-KLF4-SOX2 pathway by targeting JAK2 and SOCS3

**Fig. 3:**
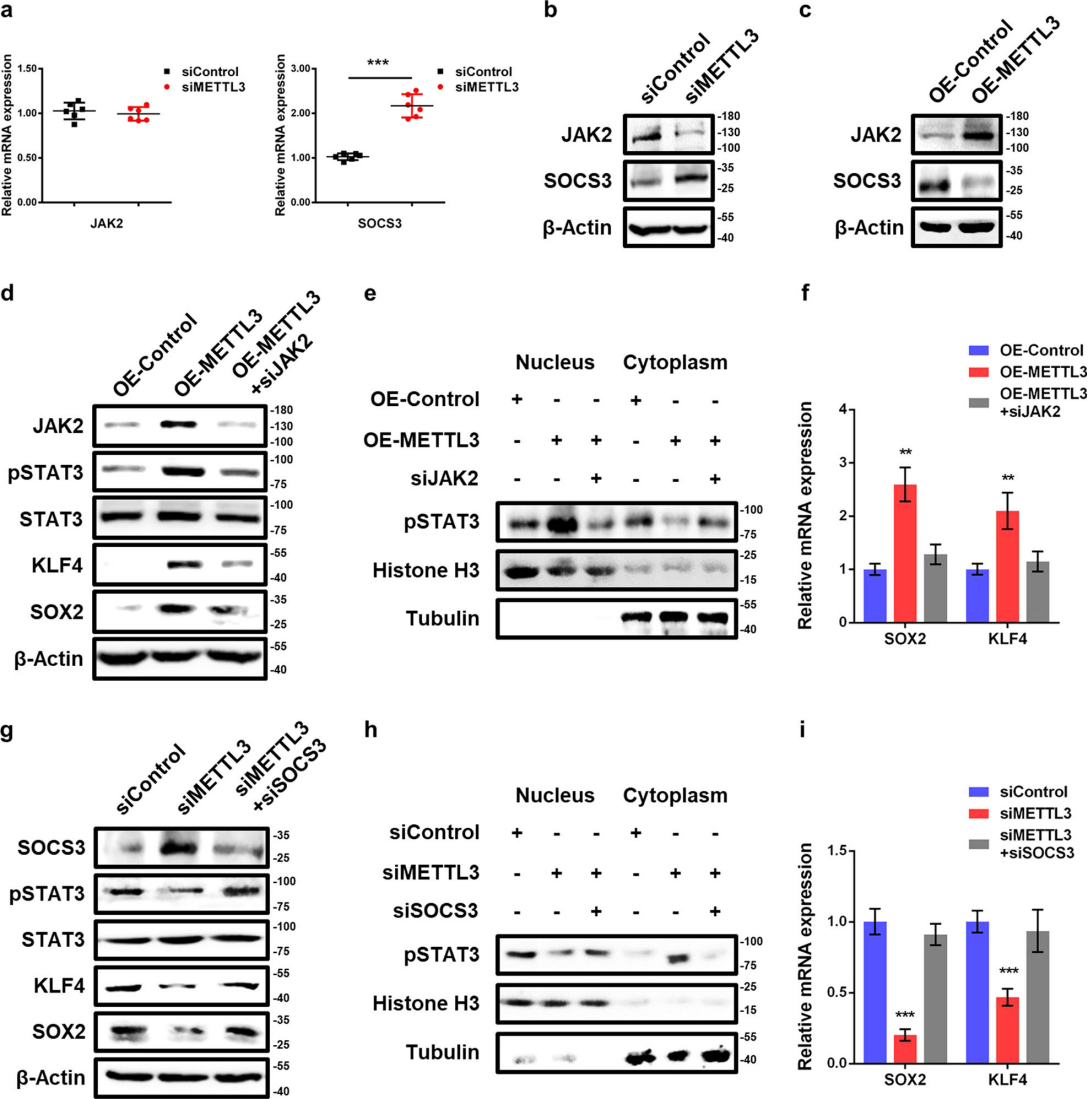
METTL3 regulates STAT3/KLF4/SOX2 pathway by mediating the expression of JAK2 and SOCS3. **a** qPCR analysis of JAK2 and SOCS3 in control and METTL3 knockdown piPSCs. GAPDH was used as an internal control. **b** Western blot analysis of JAK2 and SOCS3 in piPSCs with or without METTL3 knockdown. β-Actin was used as loading control. **c** Western blot analysis of JAK2 and SOCS3 in piPSCs with or without METTL3 overexpression. **d** Western blot analysis of JAK2, pSTAT3, STAT3, KLF4, and SOX2 in piPSCs with or without METTL3 overexpression and transfected with negative control or JAK2 siRNA. **e** Western blot of nuclear and cytoplasmic distribution of pSTAT3, KLF4, and SOX2 in piPSCs with or without METTL3 overexpression and transfected with negative control or JAK2 siRNA. **f** qPCR analysis of SOX2 and KLF4 expression in piPSCs with or without METTL3 overexpression and transfected with negative control or JAK2 siRNA. **g** Western blot analysis of SOCS3, pSTAT3, STAT3, KLF4, and SOX2 in piPSCs with or without METTL3 knockdown and transfected with negative control or SOCS3 siRNA. **h** Western blot of nuclear and cytoplasmic distribution of pSTAT3, KLF4, and SOX2 in piPSCs with or without METTL3 knockdown and transfected with negative control or SOCS3 siRNA. Histone H3 and Tubulin serve as nuclear and cytoplasmic markers, respectively. **i** qPCR analysis of SOX2 and KLF4 expression in piPSCs with or without METTL3 knockdown and transfected with negative control or SOCS3 siRNA. Data were presented as mean ± SD of three independent experiments. ^*^P < 0.05, ^**^P < 0.01, ^***^P < 0.001 compared with the control group

Previous study demonstrated that JAK2–STAT3 signaling pathway has an indispensable role in embryonic stem cell self-renewal^19^. JAK2, a non-receptor tyrosine kinase, could phosphorylate STAT3 and activate JAK2–STAT3 pathway to transduce the intracellular signal^24^. SOCS3 is a key negative regulator of JAK2–STAT3 signaling pathway and has an important role in stem cell self-renewal^25^. Based on the above findings, we investigated whether METTL3 affects STAT3 phosphorylation through JAK2 and/or SOCS3. Compared with control cells, the mRNA level of SOCS3 was increased in METTL3 knockdown cells, whereas JAK2 mRNA expression was unchanged (Fig. 3a). We also measured the protein expression of JAK2 and SOSC3 following METTL3 knockdown. Intriguingly, loss of METTL3 downregulated JAK2 protein abundance and upregulated SOCS3 protein abundance (Fig. 3b). Moreover, overexpression of METTL3 increased JAK2 protein abundance and decreased SOCS3 protein abundance (Fig. 3c).

To further validate whether METTL3 regulates STAT3-KLF4-SOX2 pathway and pluripotency of piPSCs by targeting JAK2 and SOCS3, we performed rescue experiment and found that knockdown of JAK2 reversed the activation STAT3-KLF4-SOX2 signaling and increased nuclear retention of pSTAT3 in METTL3-overexpressed cells (Fig. 3d, e). In addition, the increased mRNA levels of KLF4 and SOX2 in METTL3-overexpressed cells could be reversed by JAK2 knockdown (Fig. 3f) Furthermore, we observed that knockdown of SOCS3 could rescue the inhibition of STAT3-KLF4-SOX2 signaling and decreased nuclear retention of pSTAT3 in METTL3-depleted piPCSs (Fig. 3g, h). Silencing of SOCS3 also restored the gene expression of KLF4 and SOX2 in METTL3 knockdown cells (Fig. 3i), indicating that METTL3 knockdown suppressed STAT3-KLF4-SOX2 signaling by attenuating JAK2 and elevating SOCS3. Collectively, these finding indicate that METTL3 maintains the activation of STAT3-KLF4-SOX2 signal pathway by mediating JAK2 and SOSC3 to preserve piPSCs pluripotency.

### METTL3 mediates protein expression of JAK2 and SOSC3 in an m^6^A-dependent manner

**Fig. 4:**
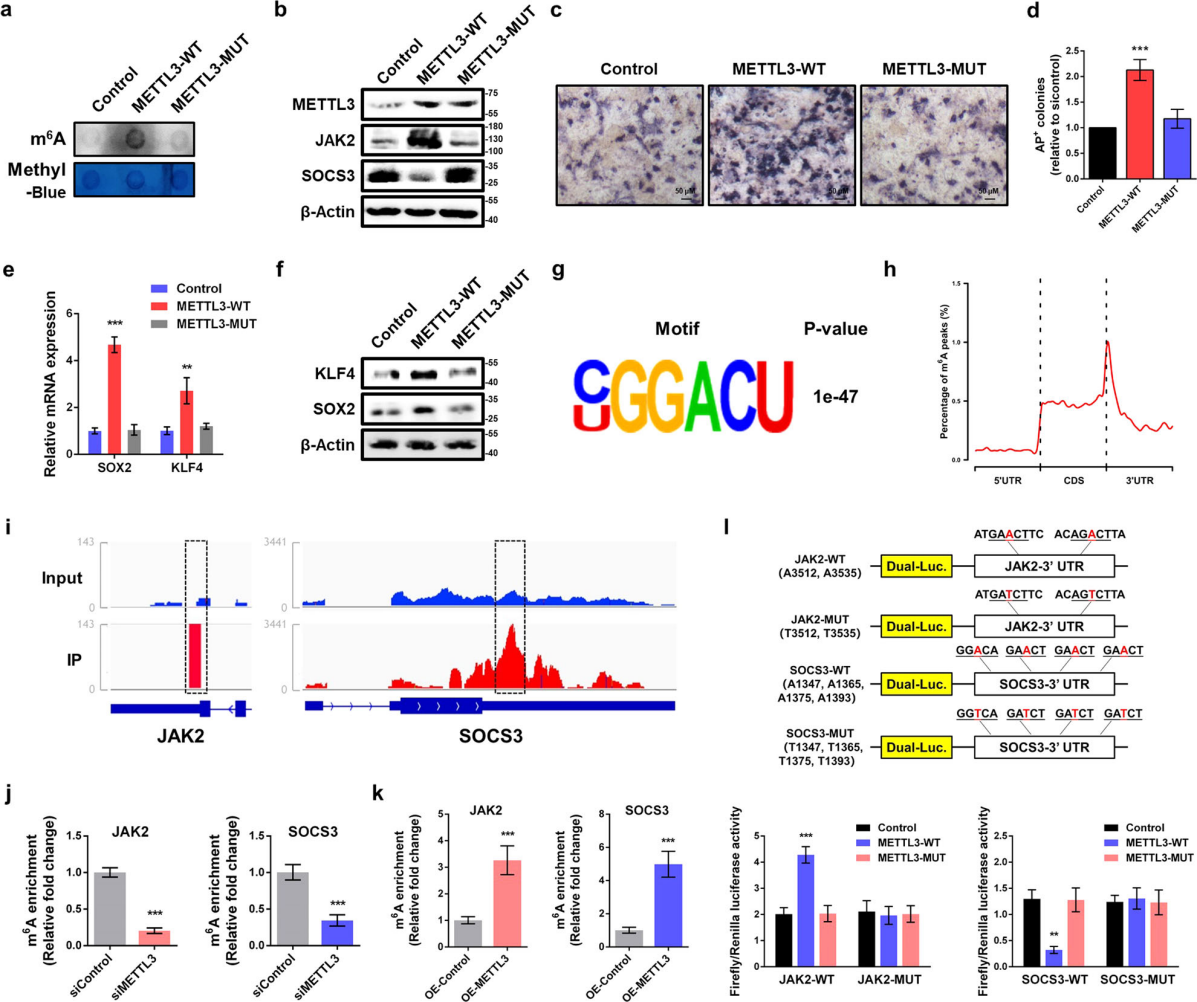
METTL3 regulates JAK2 and SOCS3 expression via m^6^A methylation. **a** m^6^A dot blot analysis in piPSCs transfected with control, wild-type (WT), and mutant (MUT) METTL3 plasmid. Equal loading of mRNA was verified by methylene blue staining (lower panel). **b** Western blot analysis of METTL3, JAK2, and SOCS3 in piPSCs transfected with control, WT, and MUT METTL3 plasmid. β-Actin was used as loading control. **c** AP staining of piPSCs transfected with control, WT, and MUT METTL3 plasmid. **d** Quantification of AP-positive colonies of piPSCs transfected with control, WT, and MUT METTL3 plasmid. **e** qPCR analysis of piPSCs transfected with control, WT, and MUT METTL3 plasmid. GAPDH was used as an internal control. **f** Western blot analysis of KLF4 and SOX2 in piPSCs transfected with control, WT, and MUT METTL3 plasmid. **g** Top consensus motif identified by HOMER with m^6^A-seq peaks in piPSCs. **h** Distribution of m^6^A peaks across the length of mRNA transcripts. Each region of 5′UTRs, CDSs, and 3′UTRs were binned into 100 segments, and the percentage of m^6^A peaks that fall within each bin was determined. **i** The m^6^A abundances in JAK2 and SOCS3 mRNA transcripts in piPSCs as detected by m^6^A-seq. The m^6^A peaks were shown in the black rectangles. **j** Methylated RNA immunoprecipitation (MeRIP)-qPCR analysis of m^6^A levels of JAK2 and SOCS3 in piPSCs with or without METTL3 knockdown. **k** MeRIP-qPCR analysis of m^6^A levels of JAK2 and SOCS3 in piPSCs transfected with control or METTL3 plasmid. **l** Relative luciferase activity of WT or MUT (A-to-T mutation) SOCS3-3′UTR (or JAK2-3′UTR) luciferase reporter in piPSCs transfected with control, WT, or MUT METTL3 plasmid. Firefly luciferase activity was measured and normalized to Renilla luciferase activity. Data were presented as mean ± SD of three independent experiments. ^**^P < 0.01, ^***^P < 0.001 compared with the control group

To explore the underlying regulatory mechanism of METTL3 on JAK2 and SOSC3 expression, we tested whether the methyltransferase activity of METTL3 is required. we first constructed plasmid to express either wild-type (METTL3-WT) or catalytic mutant METTL3 (METTL3-MUT, aa395-398, DPPW→APPW) based on published data^26^, and confirmed the effect by m^6^A dot blot (Fig. 4a). Ectopic expression of METTL3-WT, but not METTL3-MUT nor an empty vector, significantly increased the JAK2 protein abundance and decreased SOCS3 protein abundance (Fig. 4b), imply METTL3 modulated the expression of JAK2 and SOSC3 in a methyltransferase activity-dependent manner. Moreover, compared with METTL3-MUT or the empty vector, ectopic expression of METTL3-WT elevated the self-renewal ability of piPSCs (Fig. 4c, d). Consistently, the mRNA and protein levels of KLF4 and SOX2 were significantly augmented in cells expressing METTL3-WT, rather than METTL3-MUT (Fig. 4e, f). These results demonstrate that the m^6^A methylation activity of METTL3 is required for piPSCs pluripotency.

To identify and localize m^6^A sites at a transcriptome-wide level, we performed m^6^A sequencing (m^6^A-seq) to mRNA purified from piPSCs. The consensus “GGACU’’ was identified as the most enriched in the m^6^A peaks (Fig. 4g), resembling the common m^6^A motif described in mammalian cells^8,9^. Consistent with previous studies, the m^6^A peaks were especially enriched around stop codon, in 3′untranslated regions (3′UTRs) (Fig. 4h), suggesting an evolutionary conservation of m^6^A among eukaryotic species that range from human, mouse to pig. From our m^6^A-seq data of piPSCs, we found that JAK2 and SOSC3 mRNA 3′UTRs have highly enriched and specific m^6^A peaks (Fig. 4i), which is consistent with published mouse embryonic stem cell and T cell transcriptome-wide m^6^A profiling data sets^16,27^.

To ascertain whether JAK2 and SOSC3 transcripts are substrates for METTL3, we performed methylated RNA immunoprecipitation combined with qPCR (MeRIP-qPCR) to determine the JAK2 and SOSC3 m^6^A methylation levels following METTL3 knockdown. Indeed, our analysis confirmed that METTL3 knockdown decreased m^6^A levels of JAK2 and SOSC3 (Fig. 4j). Furthermore, m^6^A levels of JAK2 and SOSC3 were elevated in METTL3 overexpression piPSCs relative to control cells (Fig. 4k). More importantly, to determine whether m^6^A modifications on target mRNAs are essential for METTL3-mediated gene regulation, we performed dual-luciferase reporter and mutagenesis assays. Forced expression of METTL3-WT, but not METTL3-MUT, substantially promoted luciferase activity of reporter carrying wild-type 3′UTR fragment of JAK2, decreased luciferase activity of reporter containing wild-type 3′UTR fragment of SOCS3, relative to the control (Fig. 4l). These changes were abrogated when the m^6^A sites were mutated (A was replaced with T) (Fig. 4k). Overall, METTL3 regulates the expression of JAK2 and SOSC3, further controls pluripotency of piPSCs through m^6^A-dependent mechanism.

### Loss-of METTL3 impairs YTHDF1-mediated translation of JAK2

**Fig. 5:**
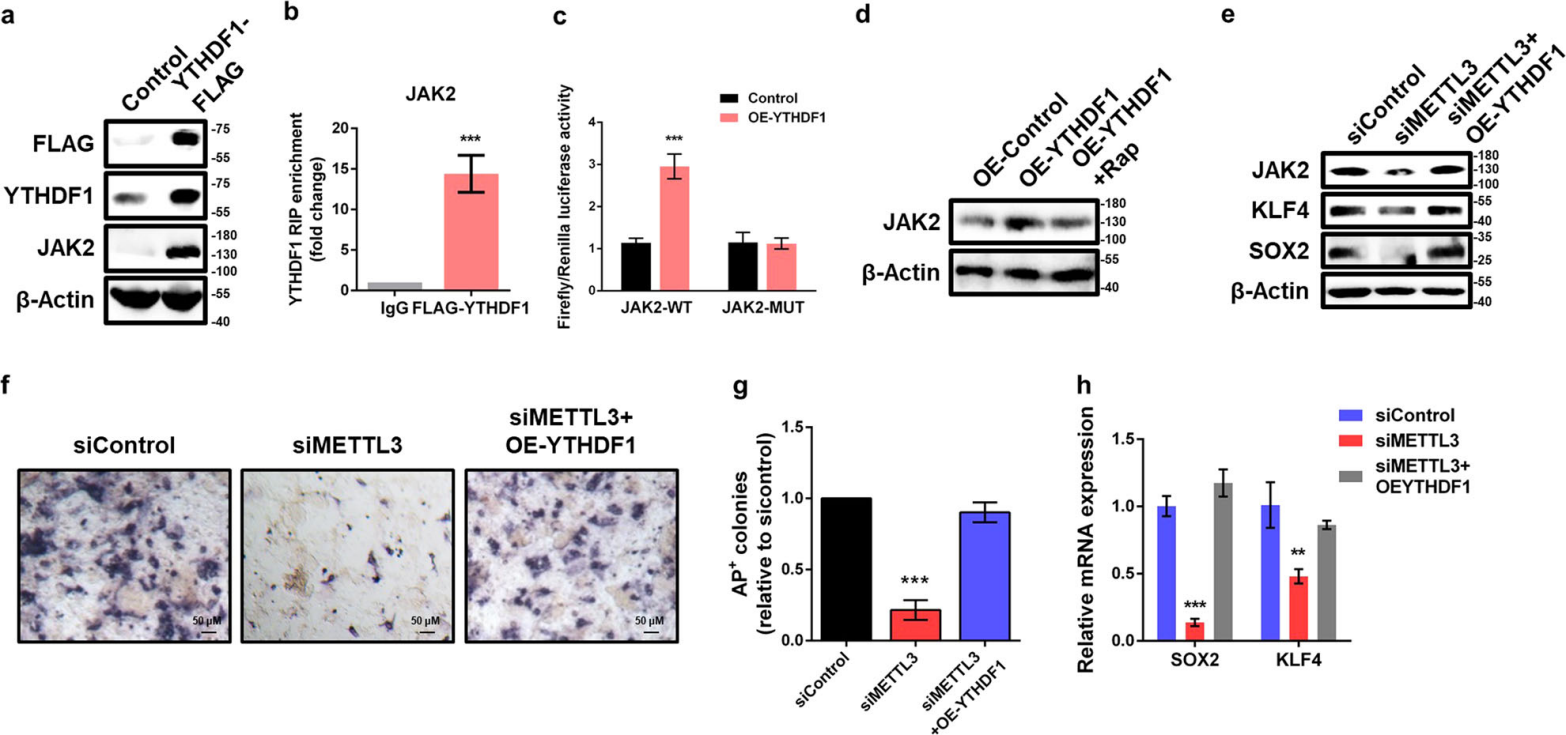
METTL3 knockdown impairs YTHDF1-mediated translation of JAK2. **a** Western blot analysis of FLAG, YTHDF1, and JAK2 in piPSCs transfected with control and YTHDF1-FLAG plasmid. β-Actin was used as loading control. **b** RIP analysis of the interaction of JAK2 with FLAG in piPSCs transfected with YTHDF2-FLAG plasmid. Enrichment of JAK2 with FLAG was measured by qPCR and normalized to input. **c** Relative luciferase activity of WT or MUT JAK2-3′UTR luciferase reporter in piPSCs transfected with control or YTHDF2 plasmid. Firefly luciferase activity was measured and normalized to Renilla luciferase activity. **d** Western blot analysis of JAK2 in piPSCs transfected with control or YTHDF1 plasmid and treated with or without 20 nm rapamycin (Rap). **e** Western blot analysis of JAK2, KLF4, and JAK2 in piPSCs with or without METTL3 knockdown and transfected with control or YTHDF1 plasmid. **f** AP staining of piPSCs with or without METTL3 knockdown and transfected with control or YTHDF1 plasmid. **g** Quantification of AP-positive colonies of piPSCs with or without METTL3 knockdown and transfected with control or YTHDF1 plasmid. **h** qPCR analysis of SOX2 and KLF4 expression in piPSCs with or without METTL3 knockdown and transfected with control or YTHDF1 plasmid. Data were presented as mean ± SD of three independent experiments. ^**^P < 0.01, ^***^P < 0.001 compared with the control group

We next explored the regulatory mechanism for how m^6^A modification regulates the expression of JAK2 and SOSC3. It is known that m^6^A should be selectively recognized by specific m^6^A-binding proteins to exerts its biological functions^7^. YTH M^6^A RNA-binding protein 1 (YTHDF1) is known to promote translation of m^6^A methylated transcripts^12^. The expression of JAK2 appeared to be promoted by m^6^A methylation, which raises the possibility that it is a target of YTHDF1. Overexpression of YTHDF1-FLAG significantly increased the protein expression of JAK2 in piPSCs (Fig. 5a), confirming that YTHDF1 is involved in regulation of JAK2. As expected, RIP-qPCR analysis revealed that JAK2 is a target gene of YTHDF1 (Fig. 5b). Moreover, Ectopic YTHDF1 significantly upregulated luciferase activity in reporters carrying wild-type 3′UTR fragment of JAK2 (Fig. 5c). Such an increase was abrogated when the m^6^A consensuses sites were mutant (Fig. 5c), suggesting an m^6^A-dependent regulation. In the case of m^6^A near stop codons or in 3′UTRs, YTHDF1 binds to select transcripts at m^6^A sites in their 3′UTRs and enhances cap-dependent translation^12^. Rapamycin, a specific inhibitor of cap-dependent protein translation, inhibits 4E-BP1 phosphorylation and causes increased association between 4E-BP1 and eIF-4E^28^. To determine whether YTHDF1 regulates JAK2 expression by promoting cap-dependent translation, we treated control and YTHDF1-overexpressed piPSCs with or without rapamycin. The results showed that rapamycin treatment markedly inhibited the increase of JAK2 protein expression in YTHDF1-overexpressed cells (Fig. 5d), indicating YTHDF1 mediates mRNA translation of JAK2 in a cap-dependent manner.

Furthermore, Ectopic expression of YTHDF1 recovered the decreased protein abundance of JAK2 in METTL3-depleted piPSCs (Fig. 5e). Overexpression of YTHDF1 could partially rescue the loss of pluripotency caused by METTL3 knockdown (Fig. 5f, g). In addition, the reduction of mRNA and protein levels of SOX2 and KLF4 were also restored by overexpression of YTHDF1 (Fig. 5e, h). Taken together, our results demonstrate that METTL3 regulates JAK2 protein expression by modulating translation in m^6^A-YTHDF1-dependent pathway.

### Knockdown of METTL3 enhances SOCS3 mRNA stability via YTHDF2-dependent pathway

**Fig. 6:**
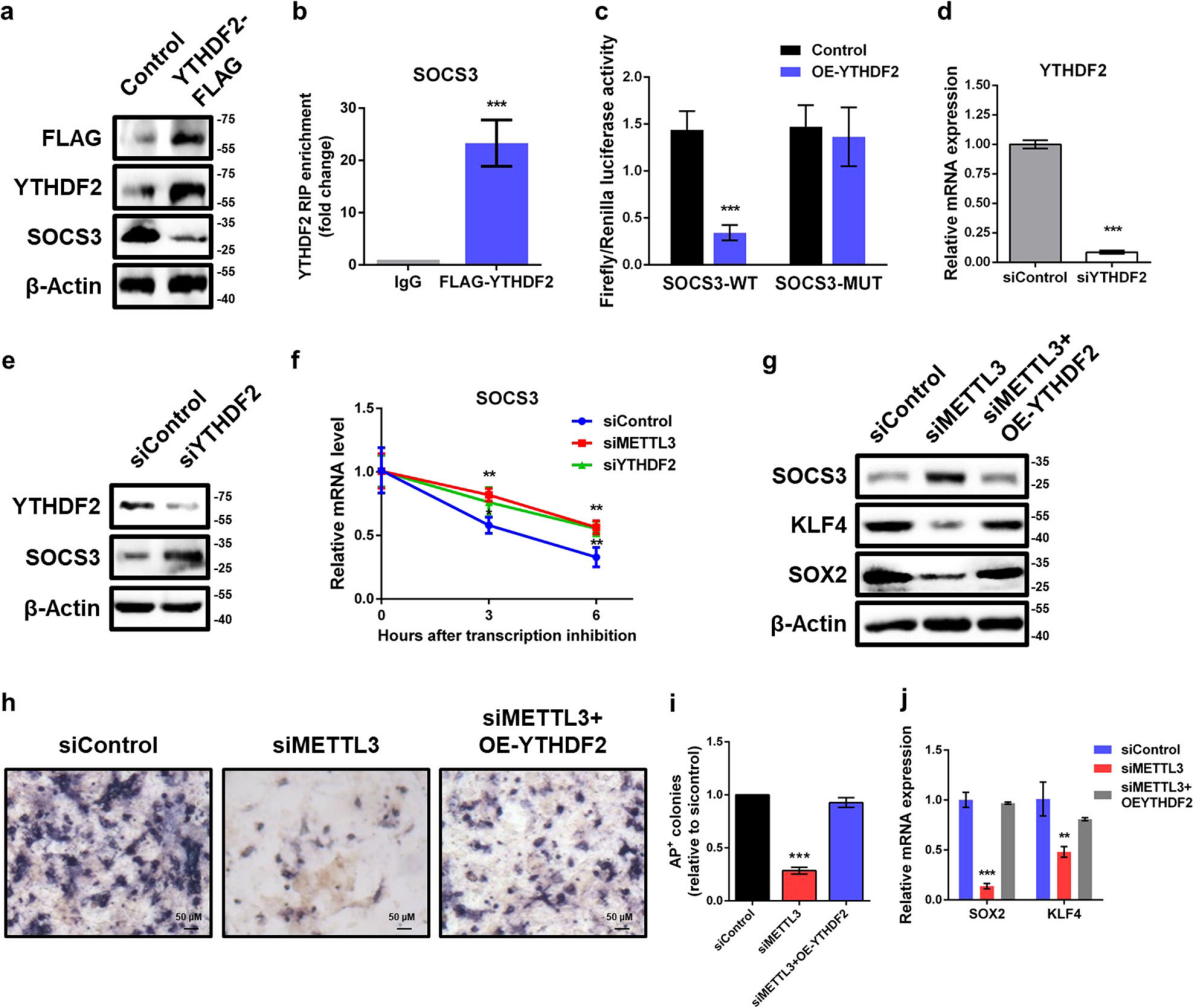
Silencing of METTL3 elevates SOCS3 mRNA stability via YTHDF2-dependent mechanism. **a** Western blot analysis of FLAG, YTHDF2, and SOCS3 in piPSCs transfected with control and YTHDF2-FLAG plasmid. β-Actin was used as loading control. **b** RNA immunoprecipitation (RIP) analysis of the interaction of SOCS3 with FLAG in piPSCs transfected with YTHDF2-FLAG plasmid. Enrichment of SOCS3 with FLAG was measured by qPCR and normalized to input. **c** Relative luciferase activity of WT or MUT SOCS3-3′UTR luciferase reporter in piPSCs transfected with control or YTHDF2 plasmid. Firefly luciferase activity was measured and normalized to Renilla luciferase activity. **d** qPCR analysis of YTHDF2 in control and YTHDF2 knockdown piPSCs. GAPDH was used as an internal control. **e** Western blot analysis of SOCS3 and YTHDF2 in piPSCs with or without YTHDF2 knockdown. **f** mRNA stability analysis of SOCS3 mRNA in control, METTL3-depleted or YTHDF2-depleted piPSCs treated with actinomycin D for 3 and 6 h. **g** Western blot analysis of SOCS3, KLF4, and SOX2 in piPSCs with or without METTL3 knockdown and transfected with control or YTHDF2 plasmid. **h** AP staining of piPSCs with or without METTL3 knockdown and transfected with control or YTHDF2 plasmid. **i** Quantification of AP-positive colonies of piPSCs with or without METTL3 knockdown and transfected with control or YTHDF2 plasmid. **j** qPCR analysis of SOX2 and KLF4 expression in piPSCs with or without METTL3 knockdown and transfected with control or YTHDF2 plasmid. Data were presented as mean ± SD of three independent experiments. ^**^P < 0.01, ^***^P < 0.001 compared with the control group

YTH M^6^A RNA binding protein 2 (YTHDF2) is reported to recognize and decay m^6^A-modified mRNA^11^. As the negative correlation between m^6^A methylation and expression of SOCS3, we hypothesized that SOCS3 transcripts might be recognized and subsequently degraded by YTHDF2. To test this hypothesis, we overexpressed YTHDF2-FLAG in piPSCs and observed a markedly decreased of SOCS3 protein levels (Fig. 6a). RNA immunoprecipitation followed by qPCR (RIP-qPCR) assay validated that SOCS3 mRNA interacts with YTHDF2-FLAG (Fig. 6b), suggesting that SOCS3 is a target of YTHDF2. Moreover, dual-luciferase assays revealed that ectopic YTHDF2 significantly downregulated luciferase activity in reporters carrying wild-type 3′UTR fragment of SOCS3 (Fig. 6c). Such a decrease was completely abrogated by mutations in the m^6^A consensuses sites (Fig. 6c), suggesting an m^6^A-dependent regulation of YTHDF2 on SOCS3 expression.

To examine the role of YTHDF2 in our system, we knocked down YTHDF2 and confirmed the knockdown efficiency by qPCR (Fig. 6d). Depletion of YTHDF2 significantly increased the protein level of SOCS3 in piPSCs (Fig. 6e). Measuring the decay of SOCS3 mRNA after blocking new RNA synthesis with actinomycin D showed that silencing YTHDF2 strikingly elevated SOCS3 mRNA stability (Fig. 6f). Similar results were also observed upon METTL3 knockdown, suggesting that YTHDF2 destabilized SOCS3 mRNA in an m^6^A-dependent manner.

Furthermore, YTHDF2 overexpression could reverse the increased protein level of SOCS3 in METTL3-depleted piPSCs (Fig. 6g). AP staining analysis suggested that adding back YTHDF2 was able to partially rescue the loss of self-renewal capacity caused by METTL3 knockdown (Fig. 6h, i). Consistently, the inhibition of SOX2 and KLF4 expression by siMETTL3 could be effectively recovered by overexpression of YTHDF2 (Fig. 6g, j). Together, these results demonstrate that YTHDF2 plays an important role in the regulation of METTL3-mediated SOCS3 expression by affecting mRNA stability.

## Discussion

**Fig. 7:**
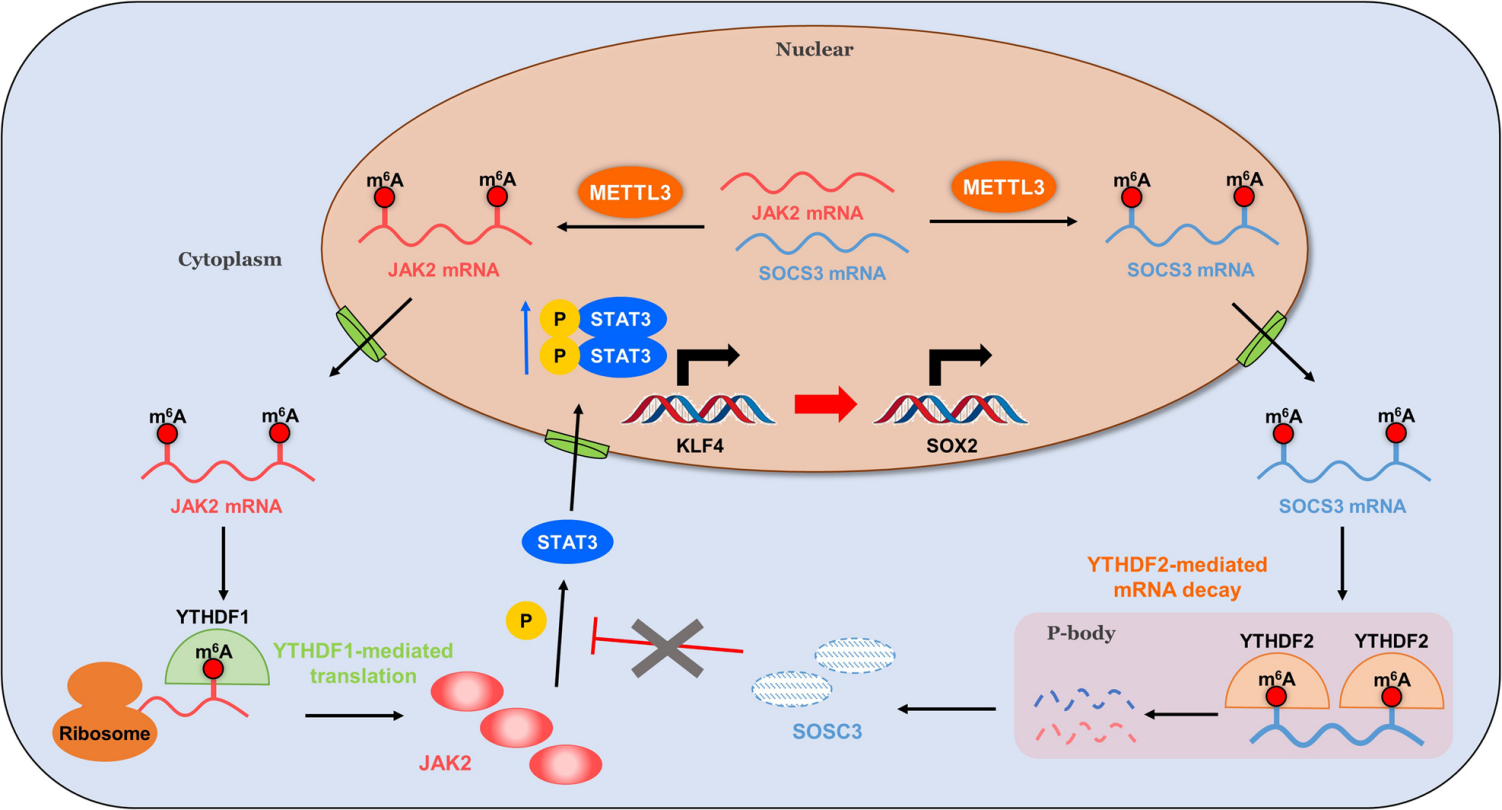
A working model summarizing the mechanism of mRNA m^6^A modification and its modulators in regulation of piPSCs pluripotency. m^6^A methyltransferase METTL3 increases the m^6^A levels of JAK2 and SOCS3 mRNA, leading to enhancing YTHDF1-mediated translation of JAK2 and attenuating YTHDF2-dependent mRNA stability of SOCS3, resulting in increased protein expression of JAK2 and decreased protein expression of SOCS3, thereby activating STAT3 phosphorylation and enhancing expression of core pluripotency genes KLF4 and SOX2 to facilitates piPSCs pluripotency

Because of the ability to infinite proliferation and give rise to all types of cells, iPSCs represent an invaluable resource to investigate human disease. Thus, in-depth understanding of the epitranscriptomic mechanisms controlling self-renewal and transitions to differentiated cell fates is essential for iPSC to hold great promise in the field of regenerative medicine^29^. Here, we identify METTL3 play a critical role in modulating piPSCs pluripotency, by mediating JAK2–STAT3 signal pathway through m^6^A-based and YTHDF1/YTHDF2-dependent post-transcriptional regulation (Fig. 7). In brief, METTL3 promotes STAT3 phosphorylation and further enhances expression of core pluripotency genes KLF4 and SOX2 by targeting JAK2 and SOSC3. METTL3 increases the m^6^A levels of JAK2 and SOSC3 mRNA, leading to enhancing YTHDF1-mediated translation of JAK2 and attenuating YTHDF2-dependent mRNA stability of SOCS3, resulting in increased protein expression of JAK2 and decreased protein expression of SOCS3, thereby activating JAK2–STAT3 pathway and facilitates piPSCs pluripotency.

Prior works had documented that m^6^A methylation has a critical role in regulation of mouse ESCs self-renewal and differentiation, the explicit function and role of m^6^A modification, however, remains controversial. Wang et al. reported that m^6^A modification on developmental regulators blocks the binding of HuR and destabilizes such transcripts, leading to maintaining pluripotency^15^. By contrast, Batista et al.^16^ demonstrated that METTL3 knockout promotes mESC self-renewal in an m6A-depedent way. Geula et al.^17^ demonstrated that depletion of METTL3 in both naive mouse ESCs and primed (epiblast stem cell, EpiSC) states resulted in upregulation of pluripotent and developmental regulators, respectively, which was explained by the fact that METTL3 targeted the dominating transcripts in either state to increase the expression of already-expressed genes. More recently, another study showed that Zc3h13 anchored the m^6^A regulatory complex in the nucleus to facilitate m^6^A methylation and mESC pluripotency^18^. Consistently, we suggest that m^6^A methylation act as a safeguard of pluripotency factors to maintains pluripotency of piPSCs, which is supported by the fact that METTL3 expression levels of piPSCs were gradually decreased during RA-induced differentiation. These studies demonstrate that the function of m^6^A methylation on pluripotency could be highly conserved between mouse and pig. Further studies are needed to confirm the extent to which the in vitro observations correlate with in vivo development.

Pluripotent cells exhibit a core transcriptional regulatory circuitry that activates stem cell-specific genes and represses developmental regulators^30^. It is well-known that JAK2–STAT3 signaling has a critical role in maintaining mESCs pluripotency by activating the downstream target KLF4 and subsequently activating SOX2^22^. Previous study reported that loss of JAK2 is lethal by embryonic day 12 in mice^31^. SOCS3 is a vital physiological inhibitor of JAK2–STAT3 signal pathway and has important roles in regulating stem cell proliferation and differentiation^32,33^. STAT3 activation is required for self-renewal of ESCs^20,21^. Leukemia inhibitory factor (LIF) signaling maintains pluripotency by inducing JAK-mediated phosphorylation of STAT3 Y705 (pY705)^34^. In agreement with these findings, we unveil that METTL3 maintains pluripotency of piPSCs by sustaining JAK2 expression, inhibiting SOCS3 expression and activating STAT3/KLF4/SOX2 signal axis. The JAK2–STAT3 pathway plays important roles in a variety of biological processes, and dysfunctional JAK2–STAT3 pathway may contribute to diseases such as cancer, heart disease and obesity^35–37^. The regulation of JAK2–STAT3 signal pathway by m^6^A methylation could be a common mechanism that affects a range of other biological processes, which should be further investigated.

The functional consequences of these dynamic and distinct RNA modifications converge mostly into regulating protein synthesis. Thus, a coordinated network of post-transcriptional modification pathways may ultimately modulate cell fate determination or stress by coordinating the mRNA stability, translation efficiency and splicing of transcripts that maintain the cell type-specific proteome. In this study, we identify that m^6^A modification regulates JAK2–STAT3 signaling in a YTHDF1/YTHDF2-orchestrated manner. Mechanistically, YTHDF1 recognizes and binds m^6^A-containing mRNA of JAK2, promotes translation and protein expression; YTHDF2 selectively targets and destabilizes m^6^A-modified mRNA of SOCS3, results in reduced protein abundance of SOCS3. Similarly, a recent study demonstrated that both of YTHDF1 and YTHDF2 were involved in regulating AKT signaling to promote the proliferation and tumorigenicity of endometrial cancer cells^38^. As m^6^A modification requires for selective recognition by specific binding proteins to exerts its biological functions^7^, other signal pathway could also be coordinately regulated by m^6^A and multiple m^6^A readers, which will be a new direction to explore in the future.

In summary, we identify m^6^A methyltransferase METTL3 as a key regulator of pluripotency and that facilitated piPSCs self-renewal. For the first time, our studies suggest that m^6^A methylation controls pluripotency by targeting SOCS3/JAK2/STAT3 signaling in a YTHDF1/YTHDF2-orchestrated manner. These results provide a better understanding of the molecular regulatory mechanisms of m^6^A methylation and its modulators in stem cell biology. The exact functions and mechanisms of m^6^A mRNA modification in iPSC pluripotency and early development are of high clinical value and certainly worth continued investigation. Ultimately, by understanding the fundamental aspects of RNA modifications we will be able to develop small-molecule inhibitors or gene therapy tools for targeting proteins that could lead to new ways of controlling gene expression or protein translation. Such discoveries might lead to the development of novel therapeutic strategies to treat complex diseases, including developmental disorders and cancer.

## Materials and methods

### Cell culture and differentiation in vitro

The mESC-like piPSCs used in this study were generated from the pig embryonic fibroblasts and provided by professor Jianyong Han^39^. These cells were maintained on mitomycin-treated mouse embryonic fibroblasts (called feeder cells) in Dulbecco's modification of Eagle medium (DMEM) supplemented with 15% serum replacement (SR) (Gibco), nonessential amino acids, l-glutamine, penicillin/streptomycin (all from Gibco, CA, USA), *β*-mercaptoethanol (Sigma, St. Louis, MO, USA), human LIF (Gibco, CA, USA), and 2i (CHIR99021 and PD0325901) (Selleck, Shanghai, China) (called 2i plus LIF medium). The medium was changed every day. To induce differentiation with RA, LIF, and 2i were removed, and RA (Sigma, St. Louis, MO, USA) was added into differentiation medium at a concentration of 5 mm. Embryoid bodies (EBs) formation was performed in “hanging drop” method as described previously^40,41^. In brief, piPSCs were digested and suspended in differentiation medium without 2i/LIF. The cell suspension was placed onto the inner surface of the lids of bacteriological grade dishes and then placed carefully in the incubator. All cells were maintained at 37°C in a humidified 5% CO_2_ incubator.

### Cell transfection, plasmids, and RNA knockdown

Cell transfection was achieved by using Lipofectamine 2000 (Invitrogen, Carlsbad, CA, USA) for plasmid and Lipofectamine RNAiMAX (Invitrogen, Carlsbad, CA, USA) for siRNA following the manufacturer’s protocols. The wild-type METTL3-CDS expression plasmid was generated by cloning the full-length ORF of pig METTL3 gene (XM_003128580.5) into pLVX vector. The catalytically mutant METTL3 (D395A and W398A) was amplified by PCR and cloned into pLVX vector based on published data^27,42,43^. Lentiviral vectors expressing METTL3 in piPSCs was purchased from Hanbio (Shanghai, China). METTL3 overexpression was achieved by lentivirus transduction in the presence of 4 µg/mL polybrene according to manufacturer’s protocols. The FLAG-YTHDF1 and FLAG-YTHDF2 expression plasmid were cloned into pcDNA3.1 mammalian expression vectors. The sequences for siRNA were listed in Table S1.

### AP staining and immunofluorescence

For AP staining, piPSCs were stained by Alkaline Phosphatase Activity Detection Kit (Sidansai Biotechnology Company, Shanghai, China) according to the manufacturer’s instructions. For immunofluorescence analysis, cells were washed with phosphate-buffered saline (PBS) and fixed with 4% paraformaldehyde for 10 min at room temperature, permeabilized with Triton X-100 for 10 min. Cells were subsequently washed with PBS three times and blocked with the immunostaining blocking buffer (Beyotime Biotechnology, Shanghai, China) for 1 h. Primary antibodies were incubated at 4°C overnight. Secondary antibodies were incubated at room temperature for 1 h. Nuclei were stained with DAPI (Beyotime Biotechnology, Shanghai, China) for 5 min at room temperature. The primary antibodies used in this work were as follows: SOX2 (1:100, sc-365964, Santa Cruz, CA, USA), pSTAT3 (1:300, ab76315, Abcam, MA, USA). The secondary antibodies used in our work were as follows: goat anti-rabbit Alexa Fluor 594 (1:500, A11037, Invitrogen, CA, USA), goat anti-mouse Alexa Fluor 594 (1:500, A11032, Invitrogen).

### Real-time quantitative PCR (qPCR)

Total RNA from the 3T3-L1 cells was extracted using TRIzol reagent (Invitrogen, CA, USA) according to the manufacturer’s protocol. cDNA was synthesized with M-MLV reverse transcriptase (Invitrogen, CA, USA) using 2 μg of extracted RNA per sample. qPCR analysis was performed using SYBR Green PCR Master Mix (Roche) with the ABI Step-One Plus^TM^ Real-Time PCR System (Applied Biosystems). GAPDH was used as an internal control. The primers used for qPCR were listed in Table S2.

### Protein extraction and western blot

Cells were washed twice with ice-cold PBS and lysed using radioimmunoprecipitation buffer lysis buffer containing a protease and phosphatase inhibitor cocktail (Beyotime Biotechnology, Shanghai, China) on ice. Equal volumes of lysates were loaded and separated by 10%–15% sodium dodecyl sulphate polyacrylamide gel electroporesis (SDS–PAGE gel and then transferred to polyvinylidene difluoride membranes. Membranes were blocked with 5% non-fat milk at room temperature for 1 h, incubated sequentially with primary and secondary antibodies with primary antibodies. The immunoblots were visualized using chemiluminescence (ECL Plus detection system). Quantification of bands was performed using Image J software. The primary antibodies used for western blot were as follows: METTL3 (1:2000, 15073-1-AP, Proteintech, IL, USA), SOCS3 (1:1000, 14025-1-AP, Proteintech, IL, USA), JAK2 (1:2000, M1501-8, Huabio, Hangzhou, China), pSTAT3 (1:2000, ab76315, Abcam, MA, USA), STAT3 (1:2000, ab68153, Abcam, MA, USA), SOX2 (1:500, sc-365964, Santa Cruz, CA, USA), KLF4 (1:2000, R1308-1, Huabio, Hangzhou, China), FLAG (1:1000, 20543-1-AP, Proteintech, IL, USA), YTHDF1 (1:1000, 17479-1-AP, Proteintech, IL, USA), YTHDF2 (1:1000, ABE542, Millipore, Darmstadt, Germany), Histone H3 (1:1000, 17168-1-AP, Proteintech, IL, USA), Tubulin (1:1000, 66031-1-Ig, Proteintech, IL, USA), β-Actin (1:1000, ab8227, Abcam, MA, USA). β-Actin was used as a loading control. The secondary antibodies used in our work were as follows: goat anti-rabbit IgG-HRP (1:3000, HA10001, Huabio, Hangzhou, China), goat anti-mouse IgG-HRP (1:3000, HA1006, Huabio, Hangzhou, China).

### Extraction of cytoplasmic and nuclear proteins

A nuclear and cytoplasmic protein extraction kit (Beyotime Biotechnology, Shanghai, China) was applied to separate these two cellular components according to the manufacturer's instructions. First, cells were harvested in cytoplasmic protein extraction buffer supplemented with phenylmethylsulfonyl fluoride (PMSF). After vortex for 5 sec and incubated on ice for 10–15 min, the cytoplasmic protein extraction buffer was added. Then the samples were incubated on ice for 5 min and centrifuged at 13,000 rpm for 5 min at 4°C. The supernatants were collected as the cytoplasmic extracts. Next, the resulting pellet was re-suspended in nuclear protein extraction buffer supplemented with PMSF and incubated on ice for at least 30 min. The resulting supernatant was gathered as nuclear extracts following centrifuge at 13,000 rpm for 10 min. The cytoplasmic and nuclear components were then subjected to Western blot.

### Analysis of m^6^A levels by LC-MS/MS

Quantitative analysis of RNA m^6^A levels by LC-MS/MS was performed as described previously^44,45^. In brief, total RNA was extracted using TRIzol reagent (Invitrogen, CA, USA), and purified using a Dynabeads mRNA DIRECT kit and RiboMinus Eukaryote Kit (Ambion, CA, USA) following the manufacturer’s instruction. About 200 ng of mRNA was digested by nuclease P1 (2 U) in 25 μl of buffer containing 10 mm of NH_4_OAc (pH = 5.3) at 42 °C for 2 h, followed by the addition of NH_4_HCO_3_ (1 m, 3 μl) and AP (0.5 U, Sigma, St. Louis, MO, USA) with incubation at 37°C for 2 h. Then the sample was diluted to a total volume of 90 µl and filtered (0.22 μm pore size, Millipore). In total, 10 µL of the solution was injected into LC-MS/MS (Agilent Technologies, CA, USA). Quantification was performed by comparison with the standard curve obtained from pure nucleoside standards. The m^6^A level was calculated as the ratio of m^6^A to A.

### Methylated RNA Immunoprecipitation coupled with quantitative real-time PCR (MeRIP-qPCR)

mRNA was prepared as described above, and fragmented using Ambion RNA Fragmentation reagent (Ambion, Carlsbad, CA, USA) at 70°C for 15 min. A small portion (10%) of the RNA fragments was collected to be used as input sample. MeRIP-qPCR was performed according to previously protocol^9^. In brief, fragmented mRNA was incubated immunoprecipitated with anti-m^6^A antibody (Synaptic Systems) in immunoprecipitation buffer (RNase inhibitor, 50 mm Tris-HCl, 750 mm NaCl and 0.5% (vol/vol) Igepal CA-630 (Sigma, St. Louis, MO, USA)) at 4 °C for 2 h with rotation. The m^6^A antibody-RNA mixture was incubated with Dynabeads protein A (Invitrogen, CA, USA) at 4°C for 2 h with rotation. The bound RNA was eluted twice by competition with M^6^A 5’-monophosphate sodium salt (Sigma, St. Louis, MO, USA) at 4°C for 1 h. Following ethanol precipitation, the input RNA and immunoprecipitated m^6^A RNAs were reverse transcribed into cDNA using M-MLV reverse transcriptase (Invitrogen, CA, USA). m^6^A enrichment was determined by qPCR analysis. The primers used for MeRIP-qPCR were listed in Table S2.

### RNA immunoprecipitation-qPCR (RIP-qPCR)

This procedure was used according to a previous published report^46^. piPSCs transfected with FLAG-YTHDF1, FLAG-YTHD2, or control plasmid were washed twice by PBS and lysed in lysis buffer of 150 mm KCl, 10 mm HEPES, 2 mm EDTA, 0.5% NP-40, 0.5 mm dithothreitol (DTT), 1 x Protease Inhibitor Cocktail and RNasin Plus RNase inhibitor (Promega, WI, USA) for 30 min at 4°C. The cell lysates were centrifuged and the supernatant was transferred to pass through a 0.45-μm membrane syringe filter. A 50-μl aliquot of cell lysate was saved as input, and the remaining sample was incubated with IgG antibody-conjugated magnetic beads or anti-FLAG magnetic beads (Sigma, St. Louis, MO, USA) for 4 h at 4 °C and six times with wash buffer (50 mm Tris, 200 mm NaCl, 2 mm EDTA, 0.05% NP40, 0.5 mm DTT, RNase inhibitor). Then the beads were eluted in wash buffer containing 0.1% SDS and 10 mL proteinase K, and incubated at 55°C for 30 min. The input and immunoprecipitated RNAs were isolated by TRIzol reagent (Invitrogen, CA, USA) and were reverse transcribed into cDNA using M-MLV reverse transcriptase (Invitrogen, CA, USA) according to manufacturer’s instruction. The fold enrichment was detected by qPCR.

### Dual-luciferase reporter and mutagenesis assays

SOCS3-3'UTR and JAK2-3'UTR with either wild-type or mutant (m^6^A was replaced by T) were inserted into downstream of pmirGLO Dual-Luciferase vector (Promega, WI, USA). For dual-luciferase reporter assay, cells seeded in 24-well plates were co-transfected with wild-type or mutant SOCS3-3'UTR (or JAK2-3'UTR) and METTL3-WT (or METTL3-MUT, or YTHDF1, or YTHDF2, or empty vector). After 48 h post transfection, the activities of firefly luciferase and Renilla luciferase in each well were determined by a Dual-Luciferase Reporter Assay System (Promega, WI, USA) according to the manufacturer's protocol.

### SOCS3-3'UTR with wild-type m^6^A sites

AGCCGGCGGGCCCAGGGGGACCACAGCAGCCTCCGCAGCGGATTCTCCTCTCCGCTTCCTCCACCCCCTGCGCTCGCAAACAGG**GGACA**CTGCGGGAGTGCT**GAACT**CGTGA**GAACT**GCCGGGAATCTTC**GAACT**TTCCAACGGAACGTGCTGGCTCTTTGATTTGGTTTAAAACAGCTTTCAACCTGAGCAGGTCTTGGGCCTG

### SOCS3-3'UTR with mutant m^6^A sites

AGCCGGCGGGCCCAGGGGGACCACAGCAGCCTCCGCAGCGGATTCTCCTCTCCGCTTCCTCCACCCCCTGCGCTCGCAAACAGG**GGTCA**CTGCGGGAGTGCT**GATCT**CGTGA**GATCT**GCCGGGAATCTTC**GATCT**TTCCAACGGAACGTGCTGGCTCTTTGATTTGGTTTAAAACAGCTTTCAACCTGAGCAGGTCTTGGGCCTG

### JAK2-3'UTR with wild-type m^6^A sites

TGAAAGAAAT**GAACT**TCATTCTGAGACCAAAAC**AGACT**TACAGAACAAAGTTTTATTTTTTACATTGCTGTAGACTACTACTGCGTACATCATTATTATGTATATCATGATGCTAGCCTGCAAAAGTATGAAAATACC

### JAK2-3'UTR with mutant m^6^A sites

TGAAAGAAAT**GATCT**TCATTCTGAGACCAAAAC**AGTCT**TACAGAACAAAGTTTTATTTTTTACATTGCTGTAGACTACTACTGCGTACATCATTATTATGTATATCATGATGCTAGCCTGCAAAAGTATGAAAATACC

### mRNA stability analysis

To determine mRNA stability, cells were treated with actinomycin D (Sigma, St. Louis, MO, USA) at a final concentration of 5 μg/mL for 0, 3, or 6 h. The cells were collected and RNA samples were extracted for reverse transcription. The mRNA transcript levels of interest were detected by qPCR^11^.

### Sequencing data analysis

The sequencing data were sent to trimmomatic to remove low quality reads and adaptor sequence contaminants under default parameters. Reads were aligned to the reference genome (Sscrofa11.1) using Tophat (v2.0.14)^47^. Gene structure annotations were downloaded from Ensemble release 94 (Sscrofa11.1). For m^6^A peak calling, the longest isoform was used if multiple isoforms were detected. The m^6^A-enriched peaks in each m^6^A immunoprecipitation sample were identified by MACS2 peak-calling software (version 2.1.1) with the corresponding input sample serving as control. MACS2 was run with default options except for ‘–nomodel,–keepdup all’ to turn off fragment size estimation and to keep all uniquely-mapping reads, respectively. A stringent cutoff threshold for Q value of 5 × 10^−2^ was used to obtain high-confidence peaks. Each peak was annotated based on Ensembl (release 94) gene annotation information by applying BEDTools’ intersectBed (v2.24.0).

### Motif identification within m^6^A peaks

The motif identification within m^6^A peaks was performed as described previously^48^. The motifs enriched in m^6^A peaks were analyzed by HOMER (v4.10.1). Motif length was restricted to 6 nucleotides. All peaks mapped to mRNAs were used as the target sequences and background sequences were constructed by randomly shuffling peaks upon total mRNAs on genome using BEDTools’ shuffleBed (v2.24.0)^49^. All piPSCs m^6^A peaks in were listed in Table S3.

### Statistical analysis

The data were presented as mean ± SD. The statistical significance of differences was determined using unpaired Student’s *t* test with GraphPad Prism 6 (Graphpad Software). *p* < 0.05 was considered statistically significant.

## Supplementary information

Supplementary Table 1

Supplementary Table 2

Supplementary Table 3
