# Supplementary material for: m6A methylation controls pluripotency of porcine induced pluripotent stem cells by targeting SOCS3/JAK2/STAT3 pathway in a YTHDF1/YTHDF2-orchestrated manner
Source: Cell Death Dis. 2019 Feb 20;10(3):171. doi: 10.1038/s41419-019-1417-4 (PMC6382841; doi:10.1038/s41419-019-1417-4)
Supplement: Supplementary file 1 — Supplementary Table 1 [file 41419_2019_1417_MOESM1_ESM.docx]

**Supplementary Table 1. RNAi oligonucleotides sequences.**

| **Gene** | **siRNA Oligonucleotides (5’-3’)** |
| --- | --- |
| Negative control | UUCUCCGAACGUGUCACGUTT |
| METTL3 | ACUUCUUCUCUAAUUCAGGGU |
| YTHDF2 | CAAGGAAACAAAGTGCAAA |
| JAK2 | GCAAAUAGAUCCAGUCCUATT |
| SOCS3 | CAAGACCUUCAGCUCCAAGTT |
