# Supplementary material for: m6A methylation controls pluripotency of porcine induced pluripotent stem cells by targeting SOCS3/JAK2/STAT3 pathway in a YTHDF1/YTHDF2-orchestrated manner
Source: Cell Death Dis. 2019 Feb 20;10(3):171. doi: 10.1038/s41419-019-1417-4 (PMC6382841; doi:10.1038/s41419-019-1417-4)
Supplement: Supplementary file 2 — Supplementary Table 2 [file 41419_2019_1417_MOESM2_ESM.docx]

**Supplementary Table 2. Sequences of qPCR primers.**

| **Gene** | **Forward primer (5’-3’)** | **Reverse primer (5’-3’)** |
| --- | --- | --- |
| **qPCR primers** |  |  |
| GAPDH | ACACTCACTCTTCTACCTTTG | GAAATTCATTGTCGTACCAG |
| METTL3 | ACACTGCTTGGTTGGTGTCA | AATCTTTCGAGTGCCAGGGG |
| YTHDF2 | CAGGCATCAGTAGGGCAACA | TTATGACCGAACCCACTGCC |
| SOX2 | CGGCGGTGGCAACTCTACTG | GGGCGAGCCGTTCATTGTAGGT |
| KLF4 | CGGACCTACTTACTCGCCTTG | CCGAACCCCAGTCAACGAA |
| NANOG | CATCTGCTGAGACCCTCGAC | GGGCTTGTGGAAGAATCAGG |
| OCT4 | CAAACTGAGGTGCCTGCCCTTC | ATTGAACTTCACCTTCCCTCCAACC |
| JAK2 | TGAGTAGGAGCCGAACCCA | ACATCTTCCCTTGCCTGCTT |
| SOCS3 | GACCAGCGACACCTCTTCAC | ATGTAGTGACGCACCAGCTT |
| PAX6 | AATTTTGCGCACACCTCTGG | GGCTAGTCCCAAGGAGGGTA |
| FGF5 | GATTCAGGCAGTCGGAGCAG | TTAGCCGAAGCGGAACTTGA |
| BRACHYURY | TACCCGACCAATCACATGCC | CTTGTGGCCTTTGCATGACC |
| FOXA2 | GATTGCCGGTCGTTTGTTGT | TGTTCATCCCGTTCATCCCC |
| GATA6 | CCTCGACCGCTTGCTATGAA | GCTGGCGTTTGTGTTGTAGG |
| **MeRIP-qPCR primers** | |  |
| JAK2 | AATCAACGCCCCTCTTTCAGA | GTACGCAGTAGTAGTCTACAGCAA |
| SOCS3 | CAGCGGATTCTCCTCTCCGC | ACCAAATCAAAGAGCCAGCACG |
